# A diagnosis of late-onset Myasthenia gravis unmasked by topical antibiotics

**DOI:** 10.1080/20009666.2018.1487245

**Published:** 2018-06-22

**Authors:** Nooreen Hussain, Faiz Hussain, Danish Haque, Subramanyam Chittivelu

**Affiliations:** a Department of Internal Medicine, University of Illinois College of Medicine at Peoria, Peoria, USA; b Internal Medicine, Advanced Cancer Care Center Illinois, Aurora, USA; c Windsor University School of Medicine, Cayon, St Kitts and Nevis; d Department of Critical Care/Pulmonary Medicine, University of Illinois College of Medicine at Peoria, Peoria, USA

**Keywords:** Myasthenia gravis, aminoglycosides, tobramycin, antibiotics, neuromuscular, eye drops

## Abstract

Myasthenia Gravis (MG) is a disorder of the neuromuscular junction (NMJ) that manifests as fluctuating fatiguable weakness of the muscles. There are many factors that can exacerbate myasthenia symptoms including a variety medications and drugs, systemic illness, and pregnancy. A number of medications have been implicated in exacerbating MG symptoms, including aminoglycosides. We present a case of an elderly female with newly diagnosed MG following the use of tobramycin eye drops for 3 days. There have been limited reports in the literature of topical medications that exacerbate MG symptoms. Clinicians prescribing tobramycin eye drops (or other associated medications) should have a high index of suspicion of MG as early discontinuation and therapy will limit long-term morbidity and mortality in these patients.

## 1. Introduction

Myasthenia gravis (MG) is a rare autoimmune disorder that affects about 5–15 per 100,000 people. MG is a disorder of the neuromuscular junction (NMJ) that manifests as fluctuating fatiguable weakness of the muscles. Symptoms manifest due to reduced binding of acetylcholine at the NMJ due to the presence of acetylcholine receptor (AchR) antibodies or less commonly, antibodies directed toward other postsynaptic skeletal muscle components, such as Muscle Specific Kinase. [1] A myasthenia crisis is a serious condition that carries a mortality rate of 3–8% [1]. Patients in a myasthenia crisis develop acute respiratory failure due to worsening MG symptoms, requiring admission to the intensive care unit. There are many factors that can exacerbate myasthenia symptoms including a variety of medications and drugs, systemic illness, and pregnancy. Infections are noted to be responsible for 40–70% of myasthenia crisis episodes [1,2].

A number of medications have been implicated in exacerbating myasthenia symptoms, including various antibiotics. Antibiotics commonly associated to exacerbating myasthenia symptoms include fluroqinolones, aminoglycosides, macrolides, and beta-lactams. [2,3] We present a case of an elderly female with newly diagnosed MG following the use of tobramycin eye drops for 3 days.

## 2. Case overview

A 73-year-old female with a past medical history of a previous cerebrovascular accident, breast cancer, and vulvovaginal adenocarcinoma was admitted for diplopia and generalized weakness. Regarding the patient’s malignancy history, she was diagnosed with advanced stage vulvar cancer 8 years previously and underwent a total abdominal hysterectomy and bilateral salpingo-oophorectomy the following year. She was diagnosed with left breast cancer the following year and underwent breast conservation surgery and radiotherapy. She was placed on anastrazole therapy thereafter and followed regularly with her oncologist. Her recent imaging studies did not demonstrate new disease.

The patient’s initials symptoms were irritation of her left eye, and after seeing her primary care provider, she was started on tobramycin eye drops (0.3% solution, every 2–3 h) for a possible ocular infection. Three days after initiating tobramycin she began experiencing diplopia, generalized weakness (primarily in the neck), and increasing respiratory distress. The patient did not recall ever having symptoms like this before and denied a history of MG. Her laboratory studies were significant for AchR-binding antibody positive at 82 nmol/L, AchR-blocking antibody positive at 56% inhibition, AchR-modulating antibody positive at 95% inhibition, and a positive striate muscle antibody titer of 1:160. A computed tomography of her chest did not show the presence of a thymoma in the mediastinum.

The patient was started on pyridostigmine therapy with improvement of her symptoms. However, while she initially responded well to pyridostigmine, she begin experiencing increasing weakness and fatigue after receiving intravenous and oral magnesium the following day, despite an increase in the dosage of pyridostigmine. She subsequently underwent six cycles of plasma exchange therapy with improvement in her symptoms however she did not tolerate nasal cannula for long periods of time. She continued to require pressure support ventilation via facemask throughout her admission. Her long-term plan was to undergo weekly PLEX therapy for 3–6 months at a long-term acute care facility, with continued ventilator support through a tracheostomy.

Unfortunately the patient’s clinical status continued to deteriorate as she developed an upper GI bleed, developed a significant anemia due to blood loss, and was intubated for respiratory failure due to prolonged periods of apnea. Shortly thereafter she went into a pulseless electrical activity cardiac arrest and underwent 30 min of cardiopulmonary resuscitation. Her family made the decision to withdraw care and she died shortly after.

## 3. Discussion

There are many medications that are known to interfere with neuromuscular transmission, and this has been seen in patients with and without a previous diagnosis of MG. [4] Fluroqinolones, aminoglycosides, macrolides, and beta-lactams are common antibiotics that have been reported to exacerbate symptoms of MG. Aminoglycosides are known to have the most potent effect on neuromuscular transmission as they exert a combined presynaptic and postsynaptic effect. [5] The drug competes with calcium for receptors and hinders the release of acetylcholine from the presynaptic membrane. By competitive inhibition it impairs the depolarization induced by acetylcholine at the postsynaptic membrane. The action potential of the myocyte membrane cannot form, which results in a blockade to the NMJ. [5] Aminoglycosides have also been described in the literature to cause a form of drug-induced myasthenic syndrome [4]. This syndrome is described as a reversible myasthenic disorder in patients with no evidence of a preexisting defect in neuromuscular transmission. Symptoms usually develop relatively soon after the drug is started. Categorizing these symptoms as a myasthenic syndrome verses newly diagnosed MG usually depends on the recovery after the medication is stopped. A myasthenic syndrome is considered when there is prompt resolution of symptoms after drug withdrawal. A prolonged recovery period after withdrawal of the medication however, is more indicative of classic MG. Our patient had a prolonged recovery after drug withdrawal and she eventually required plasma exchange therapy when her symptoms did not improve significantly with pyridostigmine. Given her clinical picture and her laboratory findings, it is likely that she had a form of late onset MG.

Patients with a generalized form of MG are classified by age of onset (earlier or later than age 50). Patients with late onset form (age of onset greater than 50) are less likely to have a thymoma, and the subtype predominately affects males. Elderly patients are also more likely to have autoantibodies against striated muscle proteins. This form is usually more severe and patients may frequently experience bulbar signs and severe respiratory crises. [6] In the early onset form (age of onset less than 50), patients are known to have other autoantibodies and can develop other autoimmune diseases such as thyroiditis. Our patient was diagnosed with late-onset MG due to her age of diagnosis and her laboratory studies being positive for AchR antibodies and her titer of striate muscle antibodies being elevated. It is well documented in the literature that the presence of striation antibodies is associated with severe disease in all MG subgroups. [7–9] This is consistent with her clinical picture as she required prolonged plasma exchange therapy and respiratory support during her course.

While it is well known that aminoglycosides can exacerbate MG symptoms, there are very limited case reports in the literature that discuss topical medications that exacerbate symptoms. We report this rare case to highlight the use of tobramycin eye drops as a precipitant of a myasthenia crisis and to characterize a form of late-onset MG in our patient who was newly diagnosed with MG at age 73. Clinicians prescribing tobramycin eye drops (or other associated medications) should have a high index of suspicion of MG as early discontinuation and therapy will limit long-term morbidity and mortality in these patients.
